# A finite element analysis on the execution effects of two novel distalization sequences in clear aligners

**DOI:** 10.3389/fbioe.2025.1664917

**Published:** 2025-11-03

**Authors:** Hongcheng Xing, Hui Li, Dongliang Zhang

**Affiliations:** ^1^ Beijing Stomatological Hospital, Capital Medical University, Beijing, China; ^2^ Capital Medical University, Beijing, China; ^3^ Beijing Engineering and Technology Research Center for Medical Implants, Beijing, China

**Keywords:** FEA (finite element analyse), clear aligners, invisalign, V-pattern sequence, molar distalization

## Abstract

**Objective:**

Current clear aligners have the problem of long treatment duration, and the effect of traditional molar distalization sequences is not ideal. To address these issues, this study proposes a new molar distalization sequence. It uses finite element analysis to simulate the long-term movement of teeth, comparing the effectiveness of the new sequence with traditional ones in moving molars backward.

**Methods:**

A standard dental arch model with normal shape was selected, and the same target positions were set. Three different movement sequences were simulated: V-pattern (21stages), ZC (abbreviation of researchers Zhang and Chang; 22 stages), and CZC (an upgraded version of ZC; 17 stages). The final positions of teeth after long-term movement were calculated, and the 3D movements (including translation and rotation) of teeth from their initial positions to the final positions in each sequence were analyzed and compared with the preset target positions.

**Results:**

The comparison showed that the final tooth positions in the CZC sequence were closest to the target positions, followed by the ZC sequence, while the V-pattern had the largest deviation from the target.

**Conclusion:**

The CZC sequence proposed in this study can shorten orthodontic treatment time and improve treatment effectiveness, providing a new idea for the design of molar distalization sequences.

## 1 Introduction

Clear aligner orthodontic technology has a history of nearly 30 years (Cong et al., 2022). Numerous factors influence the efficacy of invisible orthodontics, but the design of tooth movement sequences (i.e., staging) is currently regarded as a critical determinant (Martínez-Lozano et al., 2024). However, due to methodological challenges and limitations in studying this factor, research comparing the effects of different staging approaches remains limited. Despite this, the importance of staging design has garnered increasing attention, with recent studies increasingly focusing on its optimization (Mehta et al., 2021).

Currently, V-pattern sequences are the most widely used in invisible orthodontics, particularly for the treatment of Class II and III malocclusions that require molar distalization to gain space for subsequent corrective procedures (Kuncio et al., 2007). Invisalign predominantly employs this sequence for distalization cases. In such cases, the movement trajectory of all teeth, as displayed in the ClinCheck software staging editor, forms a V-shape (Figure 2A). However, recent clinical studies reveal limitations in the accuracy of Invisalign treatment. On average, only 50% of planned tooth movements are achieved (L et al., 2023; Haouili et al., 2020). In Class II malocclusion cases requiring continuous distalization, post-treatment outcomes often fail to meet standards set by the American Board of Orthodontics (ABO) (Taffarel et al., 2022).

Another challenge with clear aligner therapy is its prolonged treatment duration (Kuncio et al., 2007). Invisalign treatment cycles can extend up to 4.78 years, with each aligner worn for 2 weeks (approximately 400 h), requiring 20–22 h of daily wear. Adjustments during treatment may introduce delays of at least 2 months (Thukral and Gupta, 2015). Given these issues, there is a pressing need to refine the existing V-pattern sequence to improve efficacy and efficiency.

In this study, we developed a novel distalization strategy based on the traditional V-pattern sequence, which transforms the conventional single V-shaped design into two specific implementation sequences: multi-V configurations (referred to as ZC sequence) and combined V-shapes (referred to as CZC sequence). Specifically, the ZC sequence follows the movement pattern: 7-6-5-47-36-25-147-36-25-147-cycle, while the CZC sequence operates according to: 7-6-57-46-357-246-1357-246-1357-cycle.This study aims to evaluate the effectiveness of this strategic improvement through numerical simulation, so as to determine the necessity of further research on the ZC and CZC sequences. Notably, the ZC and CZC sequences have already been filed for a Chinese patent.

## 2 Materials and methods

### 2.1 Establishment of standard dentition three-dimensional model

Based on the DICOM files acquired from the CBCT scan of the standard human dental-jaw simulation model, the two-dimensional tomographic data of the mandibular dentition were obtained. Data extraction was performed using Mimics 16.0 software to reconstruct crown and root models, which were exported as STL files. These files were then processed in Geomagic Studio 2014 for repair, noise reduction, and surface optimization, followed by exportation as solid models. The solid models were imported into Hypermesh 13.0 for meshing, generating BDF-format files for subsequent finite element mesh refinement, force calculation, and analysis under different computational scenarios in specialized software.

The periodontal ligament structure was created by modifying, reconstructing, and generating thickness from the surface mesh extracted from the root regions. The orthodontic appliance structure was simulated through vacuum thermoforming membrane numerical modeling, with no overcorrection considered during appliance generation. By assembling the generated appliance model, periodontal ligament model, and tooth model (crown and root) and binding corresponding nodes, a three-dimensional biomechanical geometric model of the dentition was ultimately established. Based on the shape and geometric characteristics of each tooth, the three axes were defined for teeth with different tooth positions, and the method of definition was referred to Cong et al. (2022).

### 2.2 Material properties and meshes

**FIGURE 1:**
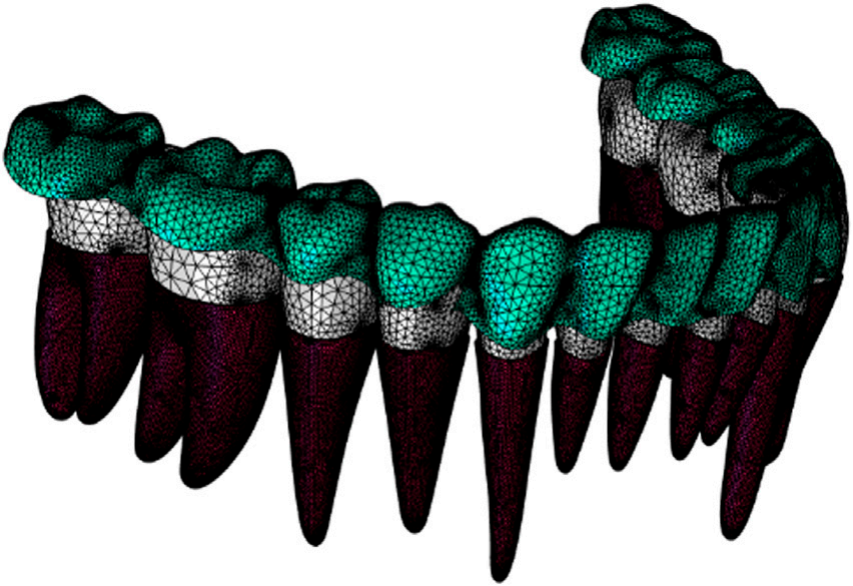
Finite element mesh model.

In order to select a suitable material constitutive model of periodontal membrane, a standard mandibular mesial incisor model was used as the research object, and a model containing teeth, periodontal membrane, and simplified alveolar bone was established, and three constitutive models, namely, Linear Elasticity, Ogden Hyperelasticity, and Reduced Polynomial Hyperelasticity, were selected to simulate the mechanical behaviors of the periodontal membrane, respectively, and compared with literature (Yoshida et al., 2001). The data in the center is compared and validated. Refer to Appendix 1 for the specific validation process. The final material properties are set as shown in Table 1 The mesh type is second order tetrahedral cell. In order to determine the optimal number of meshes, the number of 187634, 265622 and 387983 meshes were tried in the process of establishing the mesh, and after trial and error comparison, the number of 265622 meshes was found to be more applicable, based on which, further refinement of the mesh had a very small impact on the results, and the error was negligible. Therefore, after grid-independent analysis, the whole 3D structure is discretized into 265622 meshes. The grid model is shown in Figure 1 (Wang et al., 2022; Ma and Li, 2021; Wu et al., 2009; Ogden, 1973).

**TABLE 1:** Materials properties.

	Elastic modulus (MPa)			Poisson’s ratio			Mesh type		
Teeth (Wang et al., 2022)	1.96*104			0.3			C3D10M		
Clear Aligner (Ma and Li, 2021)	528			0.36			C3D10M		
PDL	Ogden Model (Wu et al., 2009; Ogden, 1973)								
	α1	μ1	α2	μ2	α3	μ3	D1	D2	D3
	24.4	1.99	15.8	3.99	8.56	2	4.87	4.87	4.87

Reference to Hamanaka et al. (2017) is made to the proposed method to accomplish the process of simulating long-term tooth movement. The prediction of tooth position after simulating multiple sets of teeth wear can be achieved by this method.

### 2.3 Three methods of tooth movement sequences

**FIGURE 2:**
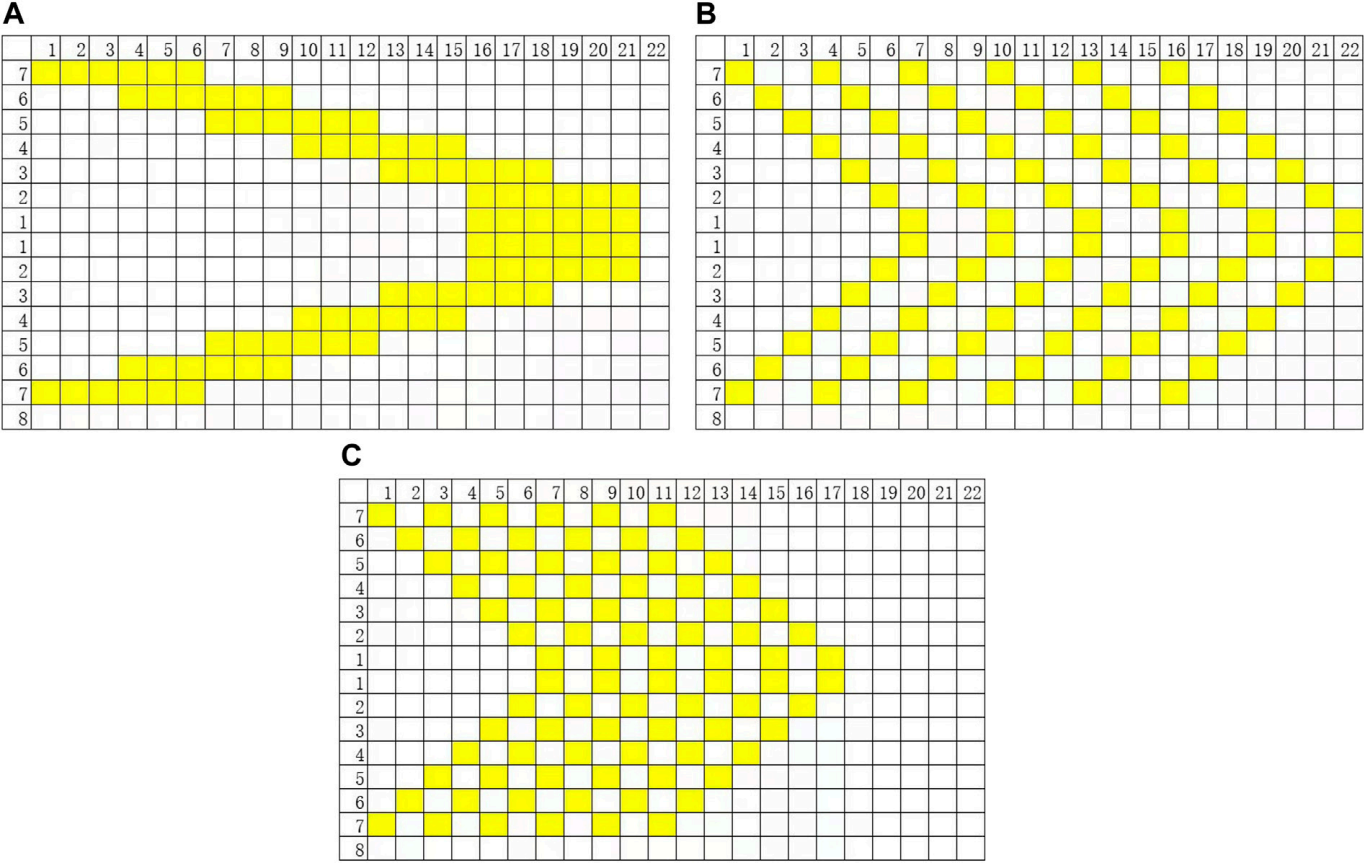
Three sequence methods: Horizontal axis = “Treatment Stages”; Vertical axis = “Tooth Position Number”. **(A)** V-pattern sequence. **(B)** ZC sequence. **(C)** CZC sequence.

The movement patterns and key parameters of the three sequences are graphically illustrated in Figures 2A–C, where the horizontal axis represents “Treatment Stages” and the vertical axis indicates “Tooth Number,” allowing for intuitive observation of the movement status of each tooth at different time points under each sequence.

In this study, iterative finite element simulations were conducted to model tooth movement using the V-pattern, ZC, and CZC sequences. The total planned distal movement for the entire arch was set at 3 mm in all three groups, with identical initial and target positions for all teeth. By controlling the single variable of “movement sequence design,” differences among the approaches were compared. Furthermore, the simulation focused exclusively on translational distal movement and did not incorporate other types of tooth movement such as torque adjustment, rotation, or tipping, so as to specifically evaluate the influence of different sequencing strategies on the accuracy and efficiency of distalization.

### 2.4 End position analysis

The final results of the three sequences of tooth movement and the initial position were calculated to obtain the amount of movement of each tooth, and the difference between the design end position and the initial position was evaluated, and the smaller the difference, the closer it was to the design target position.

## 3 Results

After validation, this model showed good consistency with the reference data in literature (Yoshida et al., 2001) and was deemed suitable for subsequent calculations. Notably, this study is a deterministic finite element simulation, without random variation within groups, so the traditional statistical significance test is not carried out.

### 3.1 Calculations of three sequences simulating long-term movements

**FIGURE 3:**
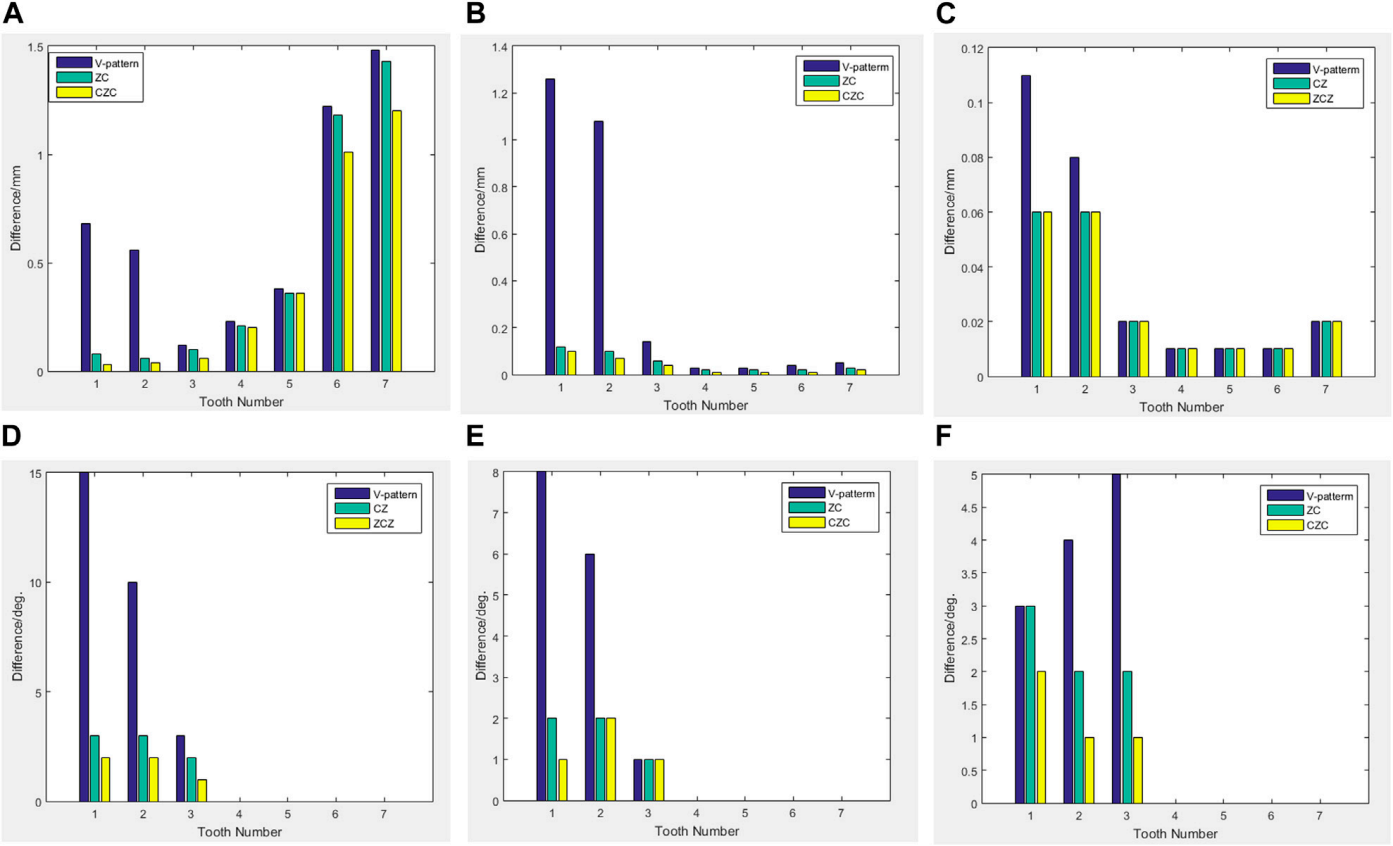
The difference between the three rotational components. **(A)** Translational component:MD. **(B)** Translational component:FL. **(C)** Translational component:OG. **(D)** Rotational component:TIP. **(E)** Rotational component:Torque. **(F)** Rotational component:Rotation.

The results from the 1st, 5th, 8th, 12th, 17th, and final steps in the long-term movement sequences were selected and presented in Table 2. Given the inherent symmetry of the data, we decomposed the motion of the right mandibular teeth numbered 1 to 7 into six-degree-of-freedom components, listing the subtraction between each motion component and its corresponding target position in Figures 3A–F. Comparative analysis revealed that among all motion component deviations from the target positions, V-pattern exhibited the largest discrepancy, while CZC showed the smallest difference.

**TABLE 2:** Long-term movement results.

Step no.	V-pattern	ZC	CZC
1	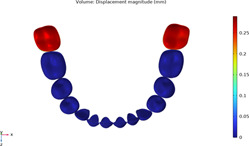	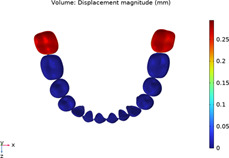	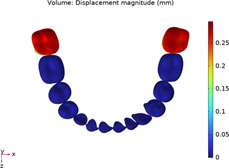
5	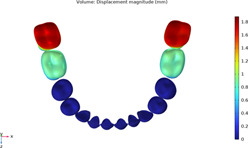	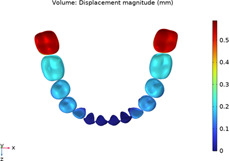	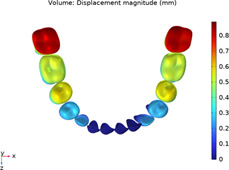
8	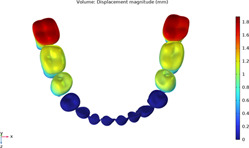	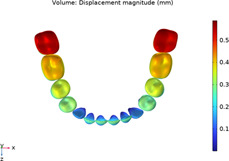	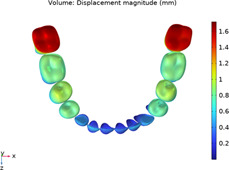
Last step	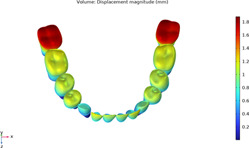	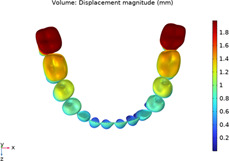	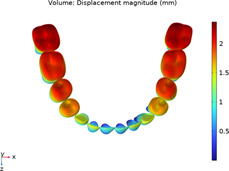
V (21)			
ZC (22)			
CZC (17)			

## 4 Discussion

### 4.1 Rationality of long-term mobility results

The algorithm for long-term tooth movement in this paper refers to the method in the literature, i.e., in the ideal way, all the initial deformations produced by the PDL are able to be finally reflected as the deformations of long-term movement. Although this method does not reflect the effect of force decay, it can be used as a good analytical tool for comparative studies (Hamanaka et al., 2017). The total amount of movement designed was 3 mm, and the current execution rates were 45.7% for V-pattern, 51.4% for ZC, and 57.1% for CZC. The results of V-pattern and ZC were compared in a previous study, and the results were consistent with the current results, with ZC being superior to V-pattern (Li and Zhang, 2024). According to the data mentioned in clinical reports (Mao et al., 2023; Crego-Ruiz and Jorba-García, 2023), the execution rate of clear aligner orthodontic technology is roughly in the range of 30%–73.8%, and the presently calculated execution rate roughly satisfies the range of execution rate mentioned in clinical reports.

### 4.2 Scientific validity of the ZC and CZC sequences

In the process of molar distalization using the conventional V-pattern sequence, anchorage loss cannot be overlooked, which manifests as mesial movement of premolars and labial movement of anterior teeth (Ravera et al., 2016; Saif et al., 2021). This unintended tooth movement not only elevates the risk of periodontal tissue complications (e.g., alveolar bone dehiscence and root resorption) but also reduces distalization efficiency due to reciprocal tooth movement (Crego-Ruiz and Jorba-García, 2023). To address these limitations, the ZC and CZC sequences, or the multi-V tooth distalization method introduced in this study were initially developed to overcome the suboptimal outcomes and prolonged treatment duration associated with conventional V-pattern sequence movement.

Although existing literature suggests that unilateral tooth movement should not involve more than two teeth per side (Daher, 2011), the present study revealed that this limitation is not absolute and depends on the specific movement strategy. Our calculations indicated that when the number of unilaterally moved teeth exceeds half of the total dentition, the efficacy of distalization is enhanced. The key to this phenomenon lies not in the number of moved teeth, but rather in whether the total counterforce meets the biomechanical requirements for tooth movement. Specifically, with a well-designed movement sequence and effective anchorage control, the maximum number of unilaterally moved teeth can reach four. This approach not only enhances anchorage expression but also significantly shortens the overall treatment course.

### 4.3 Design logic of the ZC and CZC sequences

The conventional V-pattern sequence is predicated on treating the entire dentition as a single macroscopic unit. Nevertheless, an intermediate scale exists between the whole dentition and individual teeth—we can cluster several teeth to form sub-macroscopic structures, and implement localized V-pattern sequences at this intermediate level. While preserving the overall V-pattern architecture at the macroscopic scale, this approach decomposes the long V-pattern of the entire dentition into multiple micro-V patterns corresponding to each sub-macroscopic structure.

Specifically, for the ZC sequence, the unilateral dentition is divided into three distinct sub-macroscopic structures: the anterior tooth group, premolar group, and molar group. Each sub-group follows the movement logic of the traditional V-pattern sequence independently, thereby achieving synchronized movement of three parallel micro-V patterns.

For the CZC sequence, building on the ZC sequence, the rigid boundaries between sub-groups are eliminated to allow cross-sub-group synchronized movement. For instance, after the initiation of movement in the molar group, the CZC sequence enables early activation of tooth positions in the premolar group, facilitating cross-sub-group movement combinations (e.g., molar-premolar cross-sub-group movement or premolar-anterior tooth cross-sub-group movement). This design allows more teeth to be moved simultaneously at a given time, while each sub-macroscopic structure (whether independent or cross-linked) still leverages the strong anchorage properties inherent to the V-shape. Ultimately, this configuration enhances the seating efficiency at each sub-level, accelerates the achievement of macroscopic treatment targets (owing to its synchronized movement system-like design), and theoretically addresses the issues of suboptimal treatment outcomes and prolonged treatment duration associated with the conventional V-pattern sequence.

### 4.4 Biomechanics of the ZC and CZC sequences

Existing studies have confirmed that the structural rigidity of clear aligners is significantly reduced in edentulous regions compared to dentate areas. The span of the gap covered directly affects the aligner’s force application efficiency, anchorage stability, and precision of tooth movement (Mehta et al., 2021; Baek et al., 2025; Zhang et al., 2024). When the sum of the lengths of individual teeth in the dental arch remains constant, an increase in the number of gaps within the dentition—which arises from the design of the tooth movement sequence—leads to a longer aligner segment spanning the edentulous spaces. It is therefore essential to minimize such length extension to mitigate its adverse effects.

In this study, for instance, when the V-pattern sequence was used for molar distalization, the anterior teeth had not yet started moving even after the molars had reached their target positions, such a delay led to a total extension of 3 mm in aligner length. This additional length is likely to significantly reduce force transmission efficiency. Similarly, detrimental effects associated with changes in aligner length also arise during the final stages of the V-pattern sequence, when the aligner undergoes rapid shortening as anterior teeth are retracted to close spaces. In contrast, both the ZC and CZC sequences initiate distal movement of the anterior teeth at a much earlier phase of the molar distalization process. While the distal end extends, the mesial end correspondingly shortens, thereby avoiding a significant increase in the overall length of the aligner. Specifically, the maximum increase in aligner length was only 1 mm for the ZC sequence and 1.5 mm for the CZC sequence, both of which are lower than the 3 mm increase observed in the V-pattern sequence. This reduction in edentulous span length helps minimize the adverse biomechanical effects associated with extended gap regions, thereby improving the overall efficiency of force transmission and providing a more reliable foundation for anchorage control and precise tooth movement.

### 4.5 Potential clinical advantages of the ZC and CZC sequences

The conventional V-pattern sequence has notable limitations in specific cases due to the delayed initiation of anterior tooth movement: in Class II Division 1 patients with labial inclination of anterior teeth, the weak anchorage of anterior teeth easily leads to unintended exacerbation of labial inclination during molar distalization (Owayda et al., 2024). In this study, whereas the V-pattern sequence only begins to act on the anterior region at Step 16, both the ZC and CZC sequences enable the transmission of space to the anterior region as early as Step 5. This early space transmission allows for earlier lingual uprighting of anterior teeth, which not only enhances the anchorage of anterior teeth and prevents further labial inclination during molar distalization but also reduces the mesial movement of posterior teeth when anterior teeth are retracted—an outcome particularly beneficial for Class II Division 1 patients.

Additionally, for cases with dental crowding, the V-pattern sequence’s delayed anterior tooth movement results in delayed improvement in esthetics, which may negatively affect patients’ treatment expectations and compliance (Maia et al., 2010). In contrast, by transmitting space to the anterior region at an early stage, the ZC and CZC sequences can resolve anterior crowding more rapidly and improve patients’ treatment experience.

It should be noted that to simplify the model, this study did not incorporate factors such as tooth inclination and torsion, nor did it simulate complex clinical cases. Consequently, the differences among the three sequences in improving anterior tooth uprighting and resolving crowding were not fully demonstrated.

### 4.6 Limitation

After teeth are moved under force, the traction of periodontal ligament (PDL) fibers causes teeth to have a tendency to shift back to their original positions, a phenomenon known as orthodontic relapse. Studies have shown that teeth moved by external forces are more prone to relapse if they are immediately subjected to opposite forces—i.e., when they are used as anchorage teeth right away (Edwards, 1988). This study simulated the effect of sequential molar distalization through iterative FEA; however, clinical relapse factors were not incorporated into the simulation, which may have impacted the authenticity of the results. In addition, this study did not simulate the decay process of corrective force over time, and as bone, PDL, and teeth are complex heterogeneous structures that were simplified in FEA to facilitate calculations, the FEA model may not yield conclusive results and thus requires further validation through clinical research. Furthermore, patient compliance and aligner fit also affect its real-world effectiveness. Therefore, the current FEA results still need further verification through additional studies trials, and their conclusions must be confirmed through clinical studies.

## 5 Conclusion

This study demonstrates that the proposed CZC and ZC sequences significantly improve the accuracy of molar distalization compared to the conventional V-pattern sequence, as validated through finite element-based long-term movement simulations. The CZC sequence, in particular, achieved the highest execution rate, suggesting its potential to reduce treatment duration and enhance clinical outcomes. These findings provide a biomechanical rationale for optimizing clear aligner staging strategies. Future research should focus on clinical validation of these sequences, incorporating patient-specific factors to further refine their applicability in orthodontic practice.

## Data Availability

The original contributions presented in the study are included in the article/supplementary material, further inquiries can be directed to the corresponding author.
